# *In silico* modeling of the specific inhibitory potential of thiophene-2,3-dihydro-1,5-benzothiazepine against BChE in the formation of β-amyloid plaques associated with Alzheimer's disease

**DOI:** 10.1186/1742-4682-7-22

**Published:** 2010-06-16

**Authors:** Zaheer Ul-Haq, Waqasuddin Khan, Saima Kalsoom, Farzana L Ansari

**Affiliations:** 1 Dr. Panjwani Center for Molecular Medicine and Drug Research, International Center for Chemical and Biological Sciences, University of Karachi, Karachi 75270, Pakistan; 2 Department of Chemistry, Quaid-i-Azam University, Islamabad 45320, Pakistan

## Abstract

**Background:**

Alzheimer's disease, known to be associated with the gradual loss of memory, is characterized by low concentration of acetylcholine in the hippocampus and cortex part of the brain. Inhibition of acetylcholinesterase has successfully been used as a drug target to treat Alzheimer's disease but drug resistance shown by butyrylcholinesterase remains a matter of concern in treating Alzheimer's disease. Apart from the many other reasons for Alzheimer's disease, its association with the genesis of fibrils by β-amyloid plaques is closely related to the increased activity of butyrylcholinesterase. Although few data are available on the inhibition of butyrylcholinesterase, studies have shown that that butyrylcholinesterase is a genetically validated drug target and its selective inhibition reduces the formation of β-amyloid plaques.

**Rationale:**

We previously reported the inhibition of cholinesterases by 2,3-dihydro-1, 5-benzothiazepines, and considered this class of compounds as promising inhibitors for the cure of Alzheimer's disease. One compound from the same series, when substituted with a hydroxy group at C-3 in ring A and 2-thienyl moiety as ring B, showed greater activity against butyrylcholinesterase than to acetylcholinesterase. To provide insight into the binding mode of this compound (Compound A), molecular docking in combination with molecular dynamics simulation of 5000 ps in an explicit solvent system was carried out for both cholinesterases.

**Conclusion:**

Molecular docking studies revealed that the potential of Compound A to inhibit cholinesterases was attributable to the cumulative effects of strong hydrogen bonds, cationic-π, π-π interactions and hydrophobic interactions. A comparison of the docking results of Compound A against both cholinesterases showed that amino acid residues in different sub-sites were engaged to stabilize the docked complex. The relatively high affinity of Compound A for butyrylcholinesterase was due to the additional hydrophobic interaction between the 2-thiophene moiety of Compound A and Ile69. The involvement of one catalytic triad residue (His438) of butyrylcholinesterase with the 3'-hydroxy group on ring A increases the selectivity of Compound A. C-C bond rotation around ring A also stabilizes and enhances the interaction of Compound A with butyrylcholinesterase. Furthermore, the classical network of hydrogen bonding interactions as formed by the catalytic triad of butyrylcholinesterase is disturbed by Compound A. This study may open a new avenue for structure-based drug design for Alzheimer's disease by considering the 3D-pharmacophoric features of the complex responsible for discriminating these two closely-related cholinesterases.

## Background

Alzheimer's disease (AD) or Senile Dementia of the Alzheimer Type (SDAT) is an irreversible but progressive neurodegenerative disorder caused by the loss of neurons and synapses in the cerebral cortex and certain sub-cortical regions. The main risk factor for AD is increased age: as people age, the frequency of AD increases. It is estimated that about 10% of people over 65 years of age and 50% of those over 85 suffer from AD. Unless novel treatments are developed to reduce the risk, the number of individuals with AD in the United States is expected to be 14 million by the year 2050 [1].

Cholinesterases (ChEs) are family of enzymes that share extensive sequence homology (65%). ChEs in vertebrates have been classified into two types, acetylcholinesterase (AChE) and butyrylcholinesterase (BChE), on the basis of distinct substrate specificities and inhibitor sensitivities. AChE (EC 3.1.1.7) is a key component of the cholinergic brain synapses and neuromuscular junctions. The major biological function of AChE is the termination of nerve impulse propagation by rapid hydrolysis of the cationic neurotransmitter acetylcholine (ACh). According to the cholinergic hypothesis, memory impairment in SDAT patients results from a deficiency in cholinergic function in the brain [2]. More specifically, low amounts of ACh in the hippocampus and cortex are generally considered as the cause of AD [3]. Although the exact role of BChE is not yet fully understood, it is reported to be involved in morphogenesis, cytogenesis and tumorigenesis, regulation of cell proliferation and onset of differentiation during early neuronal development, as a scavenger in the detoxification of certain chemicals, and in lipoprotein (VLD) metabolism [4]. In addition, some neuronal populations show exclusively BChE activity in the human brain [5], such as hydrolysis of ACh at CNS synapses, and replacement of AChE function in Alzheimer's brains renders BChE as a more potent drug target than AChE [6]. Biological evidence supports the role of BChE in the disruption of cholinergic neurotransmission observed in AD [7]. Processing of α-amyloid protein to β-amyloid peptide is also associated with the AD-related neurofibrillary tangles [6].

The relationship between AD and the formation of β-amyloid plaques further complicates the etiology of the disease. Many scientists believe that AD results from increased production or accumulation of α-amyloid in the brain, leading to nerve cell death. Recent research also has revealed that in the brains of AD patients, the level of acetylcholinesterase (AChE) is considerably reduced whereas that of butyrylcholinesterase (BChE) increases, thus aggravating the toxicity of β-amyloid peptide. Neurofibrillary tangles and amyloid plaques express AChE and BChE activity in AD [8]. This abnormal expression has been detected around the amyloid plaques and neurofibrillary tangles in the brains of AD patients [9]. It has also been reported that AChE and BChE co-localize within the brain in amyloid plaques to form insoluble β-amyloid fibrils [10].

Hence, the most promising therapeutic strategy for activating central cholinergic functions has been the use of cholinomimetic agents. The function of cholinesterase inhibitors (ChEIs) is to increase the endogenous levels of acetylcholine (ACh) in the brains of AD patients, eventually increasing cholinergic neurotransmission. It is not surprising that ChEIs have shown better results in the treatment of AD than any other strategy explored; for example, compounds having a 2,3,8,8a-tetrahydropyrrolo[2,3-b]indole heterocyclic system, a characteristic structural motif of alkaloids such as physostigmine and phenserine, are considered potent ChEIs for use in AD treatment [11]. Both ChEs show a characteristic cleft intruding into the enzyme surface, containing the catalytic triad and choline binding sites where ACh is cleaved. There are several structural features that delineate and differentiate the cleft between AChE and BChE, including numerous aromatic regions present in the latter but not in the former. Both ChEs have their active sites at the base of enzyme cleft of about 20 Å depth. In AChE, the binding of the substrate is represented by two phenylalanine molecules (Phe295 and Phe297) whose aromatic side chains protrude into the cleft [12]. In BChE, these two aromatic amino acid residues are replaced by two smaller amino acid residues, Leu286 and Val288. This structural difference causes a conformational change that defines a larger space in the deepest area of the cleft of BChE to allow the fitting of diverse BChEIs. The availability of BChE to catalyze diverse substrates depends on the difference between the amino acid residues that line the cleft [13]. Such structural differences allow the medicinal chemist to explore the region specific for BChE so that a combination therapy can be employed. As a drug target, it has been observed that BChE inhibition may also be more effective for the treatment of AD and related dementias [14].

**Figure 1:**
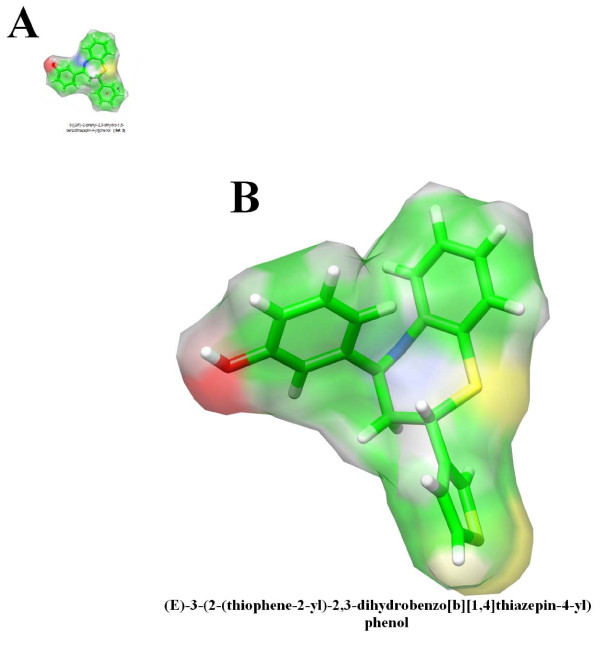
**3D View**. Energy-minimized three dimensional (3D) structure and molecular surface representation of A) symbolic compound from set 3 and B) Compound A; R_1 _= 2-thiophene moiety.

It is well established that many life-saving drugs (~30%) act by inhibiting enzymes. Therefore, the discovery of novel enzyme inhibitors has been an exciting area for pharmaceutical research leading to many interesting advances in drug development. We already have reported a number of new inhibitors of ChEs. We have performed *in vitro *testing and studied inhibition kinetics and pharmacological profiles combined with *in silico *tools, such as molecular docking and 3D-QSAR (CoMFA and CoMSIA) studies [15-24]. Benzothiazepine derivatives, two seven-membered N and S heterocyclic ring systems, have been associated with broad spectrum biological activities [25]. Continuing our ongoing research for new inhibitors of ChEs and in view of their immense pharmacological significance, we have recently synthesized a variety of 2,3-dihydro-1, 5- benzothiazepines by a [4+3] annulation of α,β-unsaturated ketones (chalcones) with *o*-aminothiophenol [26,27]. Diversity was introduced by substitutions on both rings A and B that led to the three sets of compounds: unsubstituted ring A (Set 1), 2'-hydroxy substitution on ring A (Set 2) and 3'-hydroxy substitution on ring A (Set 3). The compounds from set 1 and set 2 were generally found to be inactive. In contrast, benzothiazepines from set 3 was found to be more potent than either the unsubstituted or 2'-hydroxyl-substituted analogs, indicating that the presence of a 3'-hydroxy group on ring A may be important for inhibiting ChEs. Moreover, a benzothiazepine from the same set of compounds having a 2-thiophene moiety as ring B was found to be the most potent inhibitor of both AChE and BChE, with IC_50 _values of 5.9 and 3.97 μM, respectively (named **Compound A **in this study - Figure 1) [27]. The availability of several co-crystallized structures for both ChEs with different inhibitors makes it possible to apply a molecular docking and dynamics simulation protocol to explore the protein-ligand interactions. As mentioned earlier, the role of BChE in forming β amyloid plaques in AD patients seems to be more important, so this mechanistic study will help to predict the possible binding mode of Compound A and its dynamic behavior within the ChE active site. The study also focuses on the comparison between the inhibitory potentials of Compound A on the two ChEs. In future, it may be necessary to explore the development of potential new anti-BChE drugs for treating AD and related dementias.

## Methods

### Molecular structures

The three-dimensional (3D) structure of Compound A - (*E*)-3-(2-(thiophene-2-yl)-2,3-dihydrobenzo[*b*][1,4]thiazepin-4-yl)phenol (Figure 1) **- **was built using SYBYL^® ^software (version 7.3, TRIPOS, St. Louis, MO) [28]. Subsequently, the overall geometry was optimized by the Powell method [29] using Tripos force field [30]. 1000 iterations were given with a convergence criterion of 0.05 kcal/molÅ. Charge distributions were calculated by Gasteiger-Marsilli method [31].

### Preparation of receptor

The X-ray crystal co-ordinates of AChE (PDB ID: 1ACL) [32] and BChE (PDB ID: 1P0P) [33] in the bound state with decamethonium (DECA) and 2-(butyrylsulfanyl)-N,N,N-trimethylethanaminium (BCh), respectively, were retrieved from the Protein Data Bank (PDB) [34]. Since both ChEs have their crystal structures in a state that represents the pharmacological target for the development of new drugs to cure AD, these two PDBs were selected for modeling studies. All the heteroatoms including water molecules, bound ligands and any co-crystallized solvent were removed from the PDB file of both ChEs. It is well known that PDB files often have poor or missing assignments of explicit hydrogens, and the PDB file format cannot accommodate bond order information. Therefore, proper bonds, bond orders, hybridization and charges were assigned using the Molegro Virtual Docker (MVD - 2008, 3.2.0) [35]. Explicit hydrogens were created and their hydrogen bonding types were also determined by MVD. The potential binding sites of both ChE receptors were calculated using the built-in cavity detection algorithm implemented in MVD. The search space of the simulation exploited in the docking studies was defined as a subset region of 15.0 Å around the active site cleft.

### Molecular docking

#### Mvds docking search algorithms and scoring functions

Ligand docking studies were performed by MVD, which has recently been introduced and gained attention among medicinal chemists. MVD is a fast and flexible docking program that gives the most likely conformation of ligand binding to a macromolecule. MolDock software is based on a new heuristic search algorithm that combines differential evolution with a cavity prediction algorithm [36]. It has an interactive optimization technique inspired by Darwinian Evolution Theory (Evolutionary Algorithms - EA), in which a population of individuals is exposed to competitive selection that weeds out poor solutions. Recombination and mutation are used to generate new solutions. The scoring function of MolDock is based on the Piecewise Linear Potential (PLP), which is a simplified potential whose parameters are fit to protein-ligand structures and a binding data scoring function [37,38] that is further extended in GEMDOCK (Generic Evolutionary Method for molecular DOCK) [39] with a new hydrogen bonding term and charge schemes.

E_PLP _uses two different sets of parameters: one for approximating the steric (van der Waals) term between atoms, and the other for stronger potential for hydrogen bonds. Moreover, a re-ranking procedure was applied to obtain the highest ranked poses to increase the docking accuracy further. On average, 10 docking runs were made to obtain high docking accuracy. MolDock automatically identifies potential binding sites (cavities) using a flexible cavity detection algorithm, as there is no dependence on the orientation of the target molecule, so an arbitrary number of directions may be used. The fitness of a candidate solution is derived from the docking scoring function, E_score _and is defined by the following energy terms:

where E_inter _is the ligand-protein interaction energy:

The summation runs over all heavy atoms in the ligand and all heavy atoms in the protein, including any cofactor atoms and water molecule atoms that might be present. The second term describes the electrostatic interactions between charged atoms.

E_intra _is the internal energy of the ligand:

The double summation is between all atom pairs in the ligand, excluding atom pairs that are connected by two bonds or less. The second term is a torsional energy term, parameterized according to the hybridization types of the bonded atoms, while θ is the torsional angle of the bond. The last term, E_clash_, assigns a penalty of 1,000 if the distance between two atoms (more than two bonds apart) is less than 2.0 Å. Thus, the E_clash _term penalizes non-feasible ligand conformations.

MVD has two docking search algorithms; MolDock Optimizer and MolDock SE (Simplex Evolution). The default search algorithm used in MVD is the MolDock Optimizer [40,41], which is based on an evolutionary algorithm. From MVD version 1.5, an alternative heuristic search algorithm named MolDock SE (simplex evolution) is also implemented. MolDock SE performs better on some complexes where the standard MolDock algorithm fails. Likewise, the two scoring functions, the MolDock Score and its gird-based version, MolDock Score [GRID] [37-39], are used for evaluating docking solutions. However, exhaustive docking calculations were done using both search algorithms along with both scoring functions. The five best docking solutions were returned after each docking run. Hence, for Compound A, a total of 20 scores were generated for each PDB; however, only the results of the selected docking protocol are mentioned (see Results and Discussion). The following optimization parameters were used for individual search algorithm and scoring function.

#### Parameters for docking search algorithms

**a) MolDock Optimizer: **In MVD, selected parameters were used for the guided differential evolution algorithm: number of runs = 10 (by checking constrain poses to cavity option), population size = 50, maximum iterations = 2000, crossover rate = 0.9, and scaling factor = 0.5. A variance-based termination scheme was selected rather than root mean square deviation (RMSD). To ensure the most suitable binding mode in the binding cavity, pose clustering was employed, which led to multiple binding modes.

**b) MolDock SE: **For pose generation, 1500 maximum iterations were used by selecting a population size of 50 and were built incrementally from their rigid root point. The pose generator tests a number of different torsion angles, rotations and translations, evaluates the affected part of the molecule and chooses the value resulting in the lowest energy contribution. The poses generated were added to the population if the energy value was below the 100.0 threshold. At each step, at least 10 min torsions/translations/rotations were tested and the one giving the lowest energy was chosen. If the energy was found to be positive (owing to a clash or an unfavorable electrostatic interaction), then an additional 10 max positions were tested. If it is not possible to construct a component which does not clash, the 10 max tries number is lowered to the 10 quick try values. The Simplex Evolution parameters were set at 300 steps with neighbor distance factor of 1.0.

#### Parameters for scoring functions

**a**) **MolDock Score:** The ignore-distant-atoms option was used to ignore atoms far away from the binding site. Additionally, hydrogen bond directionality was set to check whether hydrogen bonding between potential donors and acceptors can occur. The binding site on the protein was defined as extending in X, Y and Z directions around the selected cavity with a radius of 15 Å.

**b**) **MolDock Score [GRID]: **The MolDock Score [Grid] is identical to the MolDock Score except that hydrogen bond directionality is not taken into account. The grid-based scoring function provides a 4-5 times speed-up by precalculating potential-energy values on an evenly spaced cubic grid. (Hydrogen bonding is determined solely on distance and hydrogen bonding capabilities). The energy potential is evaluated by using tri-linear interpolation between relevant grid points. The rest of the terms in the MolDock Score [Grid] version (i.e., internal ligand energy contributions and constraint penalties) are identical to the standard version of the scoring function. A grid resolution of 0.80 Å was set to initiate the docking process.

### Side chain flexibility

To account for side chain flexibility during docking in MVD, two possibilities are given: (i) flexible docking by softening potentials and (ii) indicating flexible amino acid residues during docking. The latter option was chosen for 1ACL. Trp84 and Trp279 were selected to be kept flexible during docking simulation. The repositioning of the selected side chains and minimization of ligand were performed using the standard non-softened potentials. Default potentials for Trp84 and Trp279 side chains were maintained [Tolerance = 0.9 Å, Strength = 1.0 Å, Torsional angles = 2 (each), Max T = 23.03 for Trp84 and 32.22 for Trp279, and Mean T = 16.962 for Trp84 and 19.214 for Trp279). Maximum 2000 minimization cycles (for flexible residues and ligand) along with the maximum 2000 global minimization steps were run as post-docking minimization steps using the Nelder-Mead simplex algorithm [42]. After docking of compound A into the binding pocket, the selected flexible side chains were minimized with respect to the predicted pose. After repositioning of the side chains, the ligand was subjected to further energy minimization.

The resulting docked orientations within a root-mean square deviation of 1.5 Å were clustered together. All other parameters were maintained at their default settings and the interaction mode of each pose in the active site of the receptor was determined.

### Re-docking of co-crystallized ligands

In order to develop the docking methodology, we first attempted to demonstrate that bound conformations could be reproduced *in silico*. For this purpose, DECA and BCh from the complexes 1ACL and 1P0P, respectively, were re-docked using the template docking feature implemented in MVD. The fitness evaluation of each re-dcoked pose was evaluated by considering the RMSDs values, docking scores and similarity scores. The selected re-docked pose was further evaluated by its interactions and energetic analysis to investigate the efficiency of the docking search algorithm and scoring function by comparing its values with the bound conformation.

### Molecular dynamics simulation

MD simulations of both ChEs with the docked ligand were conducted in an explicit solvent system using AMBER 9.0 package [43]. AMBER03 force field parameters were used to establish the potentials of proteins, and generalized AMBER force field (GAFF) parameters were used to establish the potentials of the inhibitor. To ensure the electro-neutrality of both complexes, seven and six sodium ions were added to the AChE and BChE systems, respectively, with subsequent solvation by TIP3P rectangular box around the solute unit. Both boxes resulted in a system of dimensions 91.759 × 89.185 × 87.051 Å^3 ^containing 6693 water molecules in AChE, and 86.344 × 85.476 × 100.642 Å^3 ^containing 7477 water molecules in BChE. Xleap was used to create the rectangular solvation box. The solvated protein-inhibitor complex system was subjected to comprehensive energy minimization before MD simulation. For this purpose, first restrain minimization of water molecules was done while holding the solute fixed (5000 steps using the steepest descent algorithm followed by 5000 steps of conjugate gradient minimizations of the whole system). This step was done to remove steric conflicts between protein-inhibitor complex and water molecules and to relax the entire system. An unrestrained minimization was then carried out using the same procedure as for restrained minimization. Bond lengths involving hydrogen atoms were constrained using SHAKE algorithm [44] with harmonic restraints of 25 kcal/molÅ. Both simulated systems were subsequently subjected to a gradual temperature increase from 0 to 300 K over 20 ps, and then equilibrated for 100 ps at 300 K followed by production runs of 5000 ps. Constant temperature (298 K) and constant pressure (1 atmosphere) were controlled by the Berendsen coupling algorithm [45] with a time constant for heat-bath coupling of 0.2 ps. The dielectric constant and cut-off distance were set to 1.0 and 10.0 Å, respectively. Long-range electrostatic calculations were carried out by particle mesh Ewald method [46]. The resulting trajectories were analyzed by PTRAJ module of AMBER package and VMD [47].

### Hardware

Docking studies were carried out on a single Intel^® ^Xeon^® ^Quad™ core processor running under LINUX OS equipped with a single user license of MVD. The molecular dynamics simulation studies were calculated using the MPI SANDER module of AMBER installed on cluster computing facility at PCMD, ICCBS, University of Karachi, consisting of 10 nodes.

## Results and discussion

### Selection of docking protocol

The selection of a valid docking protocol mainly focuses on the similarity of all re-docked poses to the crystallographically identified bound orientations. As a primary analysis measure, each docking protocol returned multiple docking poses and a symmetry-corrected RMSD was computed for all poses.

**Figure 2:**
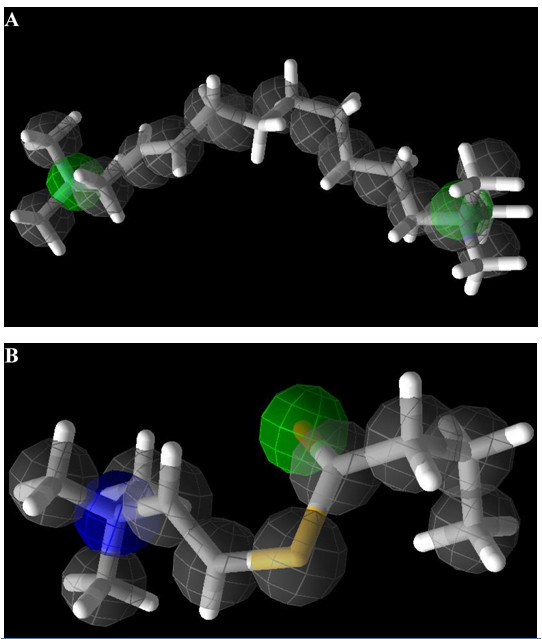
**Chemical featurers used for MVD re-docking protocol**. The chemical properties of bound ligands are shown A) DECA, Grey; Steric centers, Green; Hydrogen-bond acceptors, Blue; Positive charges and B) BCH, Grey, Steric centers, Green; Hydrogen-bond acceptors, Blue; Positive charges.

**Figure 3:**
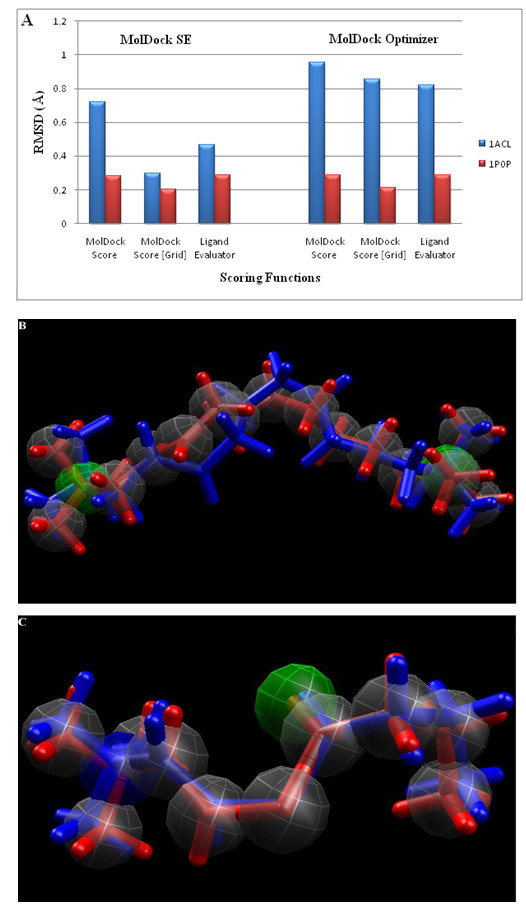
**Graphical representations of RMSD values of top-ranked re-docked poses and superimposition of selected conformations**. Re-docked poses of A) DECA and B) BCH in blue as compared to their bound crystallographic conformations in red (See also Table 1).

The chemical properties of the bound ligands that were utilized for re-docking have 18 steric centers (grey), 2 hydrogen bond acceptors (green) and 2 positive charges (blue) in DECA and 12 steric centers (grey), 2 hydrogen bond acceptors (green) and 1 positive charge (blue) in BCh (Figure 2). By using two search algorithms in conjunction with two scoring functions per 5 poses returned, 40 RMSD values (20 for each co-crystallized ligand) were obtained. An additional scoring function known as ligand evaluator was also employed, which also returned 5 poses per run. Of these, MolDock SE combined with MolDock Score [Grid] gave the lowest RMSD values for both co-crystallized ligands, that is only 0.295 Å and 0.203 Å deviations between the top-ranked poses and the experimental structures of DECA and BCh, respectively (Table 1). Figure 3 shows the graphical representation of RMSD values and the best fit re-docked co-ordinates with respect to the crystal ligand co-ordinates. A visual inspection of these poses also confirms a very good alignment of the experimental and calculated positions. These encouraging RMSD values demonstrate MVD as very accurate in reproducing the experimental binding mode.

**Table 1:** RMSD values of re-docked conformations of DECA and BCH in 1ACL and 1P0P, respectively.

Search Algorithms	Scoring Functions	RMSDs Values	
		
		PDB IDs	
		
		1ACL	1P0P
*MolDock SE*	MolDock Score	0.72	0.282
	
	*MolDock Score [Grid]*	*0.295*	*0.203*
	
	Ligand Evaluator	0.467	0.286

MolDock Optimizer	MolDock Score	0.956	0.288
	
	MolDock Score [Grid]	0.855	0.211
	
	Ligand Evaluator	0.820	0.211

Table shows only the best RMSD values of each docking run. *Italic *fonts indicate the best docking protocol (See also Figure 3)

### Evaluation of selected search algorithm and scoring function

**Figure 4:**
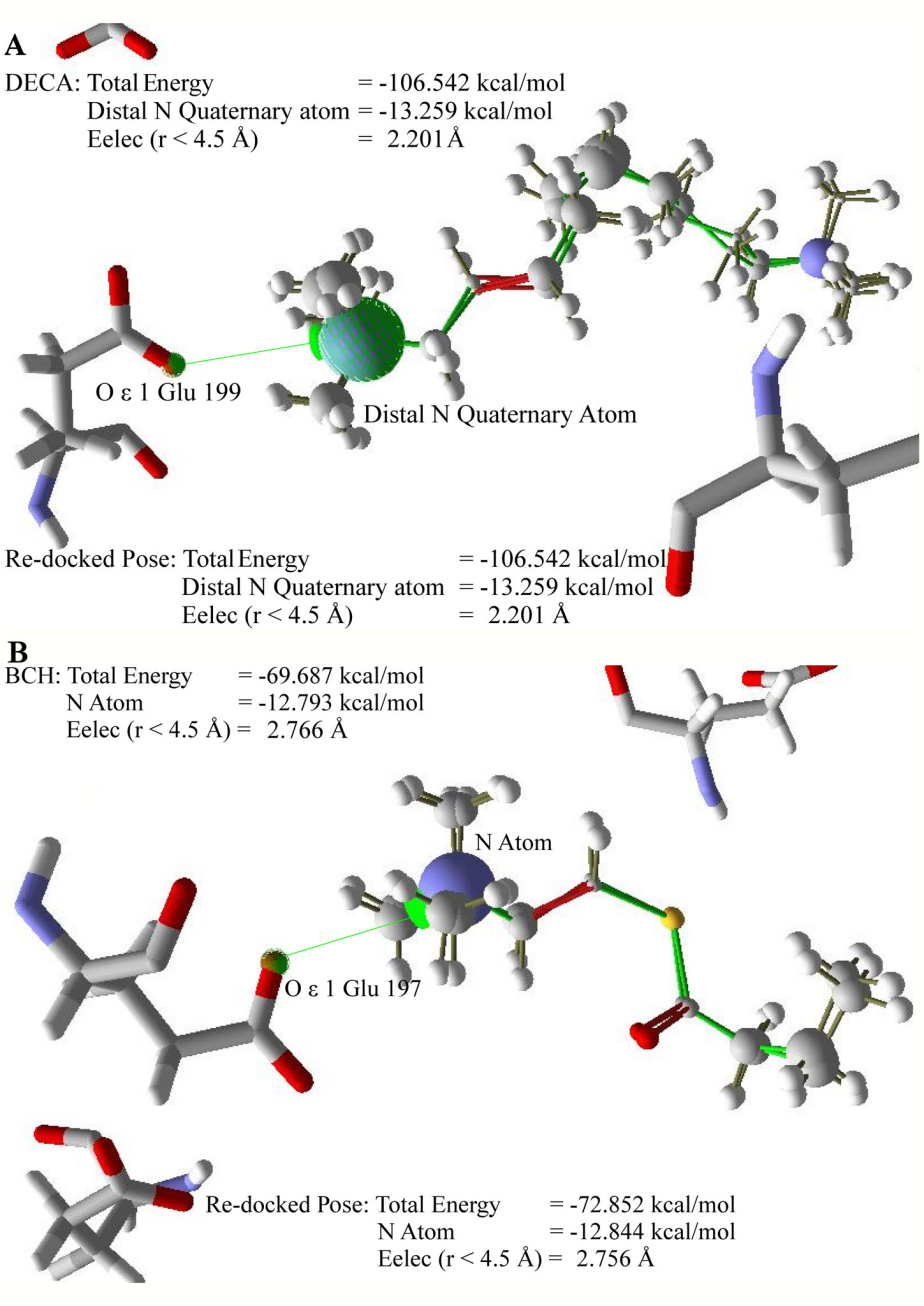
**Evaluation of selected docking protocol**. Atoms of bound and re-docked conformations are scaled according to their energy contributions. The green line denotes the electrostatic bond A) DECA - 4.341 Å (4.354 Å for re-docked pose) and B) BCH - 3.887 Å (3.879 Å for re-docked pose).

As mentioned earlier, the search algorithm MolDcok SE in combination with the scoring function MolDock Score [Grid] gives the lowest RMSD values of re-docked poses with reference to the bound crystal conformations. However, that is not the only criterion for selecting this docking protocol. Therefore, we applied a more stringent measure to ensure the selected docking protocol showed lower bias. Energetic analysis and interactions as given by the selected docking protocol of top-ranked poses having least RMSD values were compared to their respective co-crystallized ligands. In 1ACL, the total pose energy of the bound DECA was found to be -106.542 kcal/mol (-107.498 kcal/mol for re-docked pose) with the distal quaternary nitrogen atom having an energy contribution of -13.259 kcal/mol (-13.436 kcal/mol for re-docked pose). The same nitrogen atom is also involved in making long-range pair-wise electrostatic interactions [E_elec _(r < 4.5 Å)] with O ε 1 of Glu199 at a value of -2.201 kcal/mol (-2.188 kcal/mol for re-docked pose), having a distance of 4.341 Å (4.354 Å for re-docked pose). In the case of 1P0P, the total pose energy of the bound BCh was found to be -69.687 kcal/mol (-72.859 kcal/mol for re-docked pose) with the nitrogen atom having an energy contribution of -12.793 kcal/mol (-12.844 kcal/mol for re-docked pose). The same nitrogen atom is also involved in making a long-range pair-wise electrostatic interaction [E_elec _(r < 4.5 Å)] with O ε 1 of Glu197 at a value of -2.766 kcal/mol (-2.756 kcal/mol for re-docked pose), having a distance of 3.887 Å (3.879 Å for re-docked pose) (Figure 4). Other docking protocols with higher RMSD values were also checked by such analysis, but none of them provided similarity to these parameters (results not shown). Therefore, it was decided to apply this docking protocol to Compound A and to obtain the best model. The results of the same docking protocol were mentioned later (as it also gave the least MolDock Score [Grid]).

### Evaluation of poses

After selection and evaluation of the docking protocol, same docking method was applied to dock Compound A on both ChEs. The docked 3D structures of Compound A were scored, re-ranked and compared with their respective X-ray crystallographic structures. An interesting observation was that two major clusters of binding poses were found to occupy two separate but overlapping regions.

Apart from minor differences, a major diversity in the ligand-AChE complexes was the variation in the positioning of the side chain of Phe330. The divergence in the orientation of the aromatic ring of Phe330 must clearly be taken into account in designing anticholinesterse drugs. It has been found that Phe330 engages in cation interactions through its π electrons. Moreover, its side chain also guards access to the bottom of the cleft and adopts three major conformations; open, closed, and an intermediate access position [48]. For cleft-spanning ligands such as DECA, Phe330 assumes an open access position in which the side chain is shifted 3.5 Å towards the exterior and parallel to the DECA, resulting in a wider space than 1W6R (*Torpedo calcifornica *AChE complexed with galanthamine). This orientation is considered not only safer for hindrance-free entry to the ligand but also suitable to observe its experimental rationale as low activity. Therefore, the crystal structure of cocrystallized DECA was used to study protein-ligand interactions in order to control the performance of our docking approach. The active site is located 20 Å away from the protein surface at the bottom of a deep and narrow cleft [49]. For BChE, 1P0P was selected since it has the same substrate analog (butyrylcholine - BCh) as used for biological screening of BChE.

**Figure 5:**
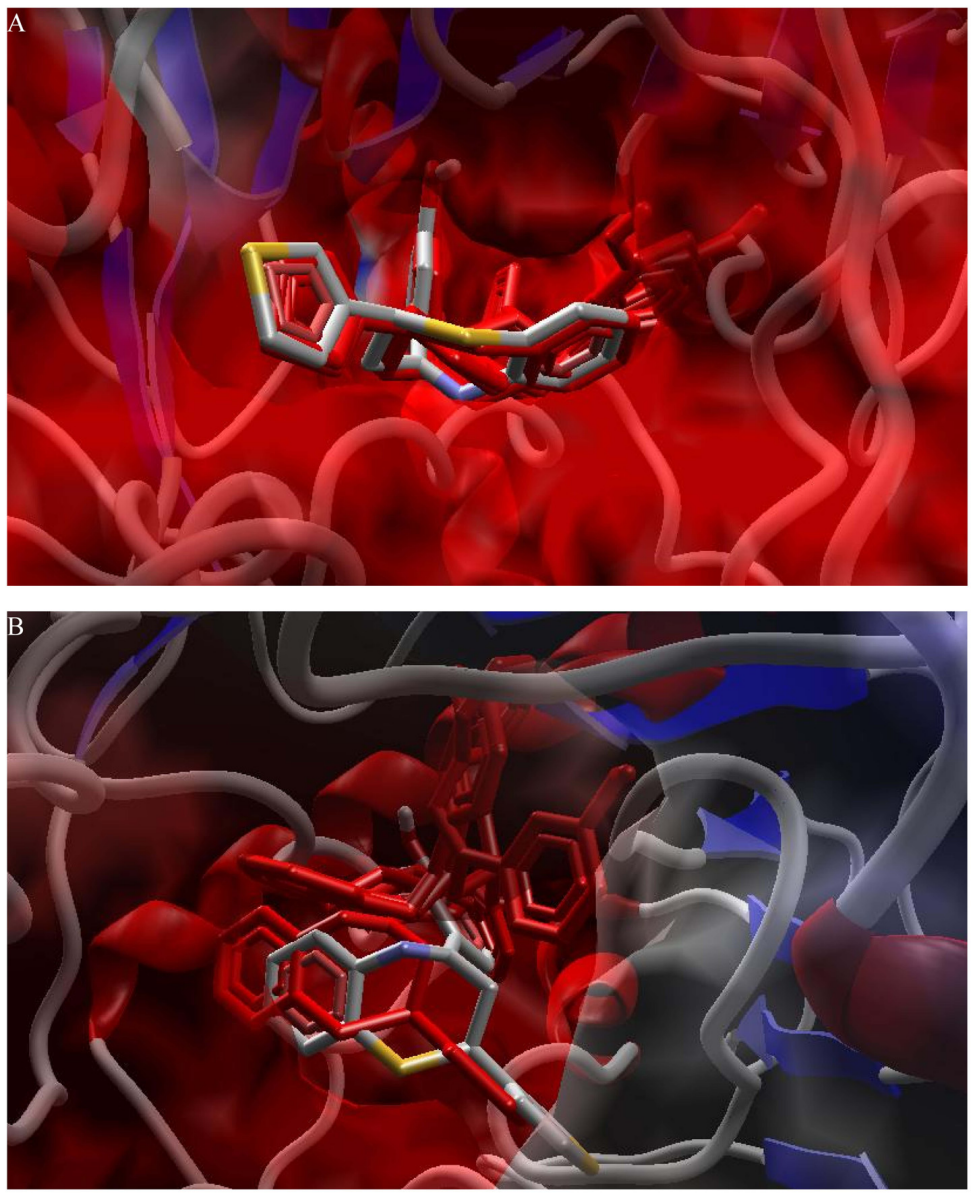
**Five Predicted binding modes of Compound A**. A) 1ACL and B) 1P0P. Rejected poses are represented as stick in red while selected pose is represented as color by element mode in stick.

**Figure 6:**
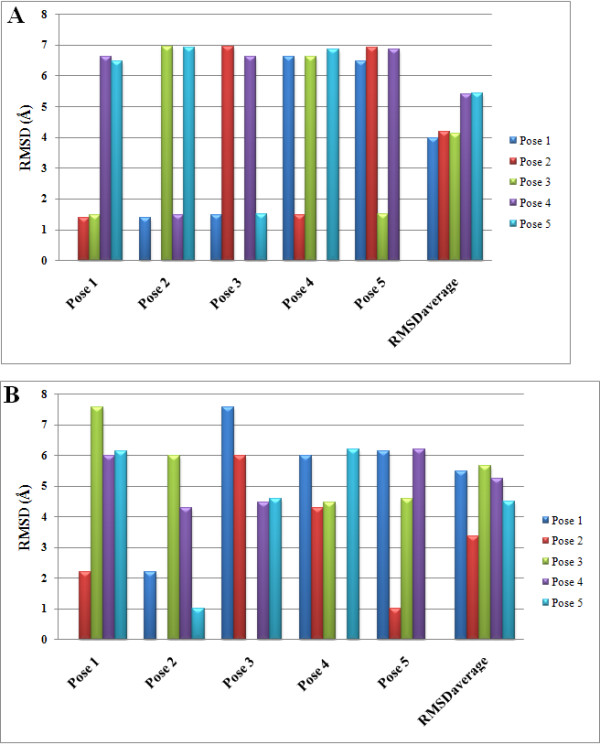
**Graphical representation of pair-wise Atom-Atom RMSD (Ǻ) (checking all automorphisms) of all 5 poses obtained**. A) 1ACL and B) 1P0P.

Figure 5 shows five docked conformations of Compound A in the aromatic cleft of AChE and in the hydrophobic pocket of BChE. Table 2 summarizes the docking results of Compound A on ChEs. The lowest MolDock scoring function (based on energy) for all five poses was found during the docking procedure, indicating that the phase space was sufficiently sampled. Since our main objective is to find the best model for Compound A, pose energies of the docked compound itself along with the analysis of molecular features and protein-ligand interactions, and the MolDock score [Grid], were selected as filtration criteria to reject other poses. Prior to this, the RMSD matrix was used to quickly inspect internal deviations between the poses themselves. Pairwise Atom-Atom RMSD (by checking all automorphisms) was taken into account to consider the inherent symmetries of the molecule when calculating RMSDs (Table 3 and Figure 6). In the case of 1ACL, pose 1 was found to have the lowest RMSD_average _value (3.987 Å) compared to its counterparts, which showed that the molecular conformation as possessed by pose 1 is a combination of all co-ordinates that other poses acquired during the docking run. It is evident from Figure 6 that pose 1 differed from pose 2 and pose 3 by 1.394 Å and 1.473 Å, respectively. Therefore, the contribution of the energy feature may also be taken into consideration for selecting pose 1 as the best model for further study. A comparison of the pose energies of conformations 1, 2 and 3 provides a sound justification for selecting pose 1 with energy -141.930 kcal/mol (-136.632 kcal/mol for pose 2 and -136.427 kcal/mol for pose 3) as the model for carrying out further modeling steps. Likewise, pose 2 and pose 4 as well as pose 3 and pose 5 have pair-wise RMSDs ≥ to 1.5 Å, but neither individual pose energies nor MolDock Score [Grid] values allowed these poses to be selected as the best model. Moreover, hydrogen bonding interaction energy, MolDock Score [Grid] and re-rank scores provide further confirmation of pose 1 as the best choice. The same is true for the docked conformations of compound A in BChE. Pose 2 gives the lowest RMDS_average _value of 3.377 Å with a conformation closely related to pose 5 (1.018 Å) and pose 1 (2.198 Å). Pose 2 and pose 5 have discrepancies in their 3'-hydroxyl group positions on ring A and both can be considered as mirror images owing to C-C bond rotation. Table 2 B shows that pose 1 has the lowest pose energy of all but its interactions with the neighboring amino acid residues are not strong enough compared to pose 2.

**Table 2:** Energetic analysis of docked Compound A on ChEs.

(A) AChE						
**Poses**	**MolDock Score [Grid]**	**E-Intra (vdw)**	**H-Bond (kcal/mol)**	**Non H-Bond (kcal/mol)**	**Pose Energy (kcal/mol)**	**Re-rank Score**

*Pose 1*	*-125.374*	*71.119*	*-4.192*	*-4.192*	*-124.1*	*-104.41*

Pose 2	-122.393	65.184	-5.026	-7.415	-123.899	-97.385

Pose 3	-122.31	75.752	-6.849	-7.294	-121.124	-92.751

Pose 4	-120.076	71.516	-2.5	-2.5	-119.209	-95.735

Pose 5	-120.037	75.257	-2.5	-2.5	-118.721	-92.9156

**(B) BChE**						

**Poses**	**MolDock Score [Grid]**	**E-Intra (vdw)**	**H-Bond (kcal/mol)**	**Non H-Bond (kcal/mol)**	**Pose Energy (kcal/mol)**	**Re-rank Score**

Pose 1	-125.374	71.119	-4.192	-4.192	-124.1	-104.41

*Pose 2*	*-122.393*	*65.184*	*-5.026*	*-7.415*	*-123.899*	*-97.385*

Pose 3	-122.31	75.752	-6.849	-7.294	-121.124	-92.751

Pose 4	-120.076	71.516	-2.5	-2.5	-119.209	-95.735

Pose 5	-120.037	75.257	-2.5	-2.5	-118.721	-92.9156

Pose 1 for AChE and Pose 2 for BChE are selected as best model for further modeling studies. Selected poses are indicated as *Italic *fonts

**Table 3:** Pair-wise Atom-Atom RMSD (Ǻ) (checking all automorphisms) of all 5 poses obtained.

(A) AChE						
**Poses**	**Pose 1**	**Pose 2**	**Pose 3**	**Pose 4**	**Pose 5**	**RMSD_average_**

*Pose 1*		*1.394*	*1.473*	*6.61*	*6.471*	*3.987*

Pose 2	1.394		6.941	1.473	6.928	4.184

Pose 3	1.473	6.941		6.61	1.51	4.134

Pose 4	6.61	1.473	6.61		6.874	5.392

Pose 5	6.471	6.928	1.51	6.874		5.446

**(B) BChE**						

**Poses**	**Pose 1**	**Pose 2**	**Pose 3**	**Pose 4**	**Pose 5**	**RMSD**_**average**_

Pose 1		2.198	7.576	5.99	6.144	5.477

*Pose 2*	*2.198*		*5.995*	*4.297*	*1.018*	*3.377*

Pose 3	7.576	5.995		4.456	4.578	5.651

Pose 4	5.99	4.297	4.456		6.205	5.237

Pose 5	6.144	1.018	4.578	6.205		4.486

Automorphisms of selected poses are indicated as *italic *fonts (See Figure 6 also)

Hence, the possible ligand-enzyme interactions of benzothiazepine are evaluated by our selected models, providing a deeper insight into the mechanism of their interactions and thus helping in the design of potent new ChE inhibitors. The interaction mode of the selected pose within the active sites of ChEs is described below.

### Compound A-AChE complex

**Figure 7:**
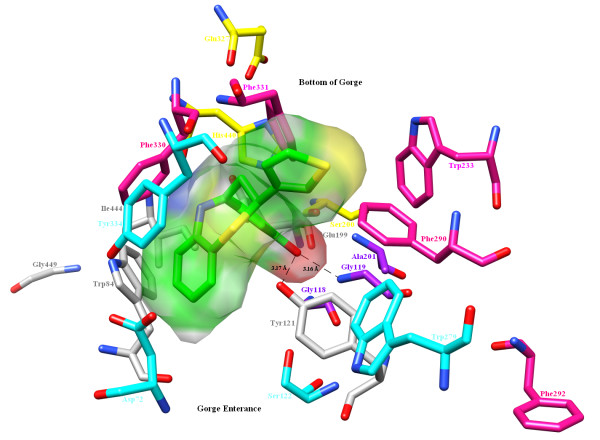
**Docked pose of Compound A in AChE**. CT (Ser200, His440, Glu327; yellow), OH (Gly118, Gly119, Ala201; purple), AS (Trp84, *Tyr121*, Glu199, Gly449, Ile444; Grey), ABP (Trp233, Phe290, Phe292, Phe330, *Phe331*; Deep pink), PAS (Asp72, *Tyr121*, Ser122, Trp279, *Phe331*, Tyr334; Cyan) and Compound A (green) are represented as stick model. The hydrogen bonding (black dashed lines with bond lengths) by flexible orientation of ring A and molecular surface of Compound A shows how well it fits in the deep and narrow aromatic gorge lined with aromatic residues. The residues indicated as *italic *fonts in legend are considered as dual characteristics.

The active site of AChE is subdivided into several subsites; for example, the esteratic subsite, also called the catalytic triad (CT, Ser200, His440, Glu327), oxyanion hole (OH, Gly118, Gly119, Ala201), anionic subsite (AS, Trp84, Tyr121, Glu199, Gly449, Ile444), acyl binding pocket (ABP, Trp233, Phe288, Phe290, Phe292, Phe330, Phe331) and peripheral anionic subsite (PAS, Asp72, Tyr121, Ser122, Trp279, Phe331, Tyr334) are buried at the bottom of a 20 Å deep aromatic cleft [50]. No interaction was observed with the atoms of the catalytic triad. The O γ of Ser200 is located 4.8 Å, N ε 2 of His440 5.8 Å and O ε 1 of Glu327 10.4 Å away from the 3'-hydroxy group on ring A. However, the peptidic NH of Gly119 in the oxyanion hole is involved as a donor in hydrogen bonding interaction with this 3'-hydroxy group. Due to this interaction, the π-electrons of the peptide bond are more delocalized, further weakening the peptide bond, and may disorientate the 3D structure of AChE. The hydroxy substituent on ring A also mediates another hydrogen bonding interaction with the η hydroxy group of Tyr121 of AS. This hydrogen bond is approximately equivalent in length (3.17 Å) to the previous hydrogen bond with Gly119 (3.16 Å). Energetically, the hydrogen bonding with Tyr121 is more favorable (-1.92 kcal/mol) than that with Gly119 (0.79 kcal/mol), as shown in Figure 7.

**Figure 8:**
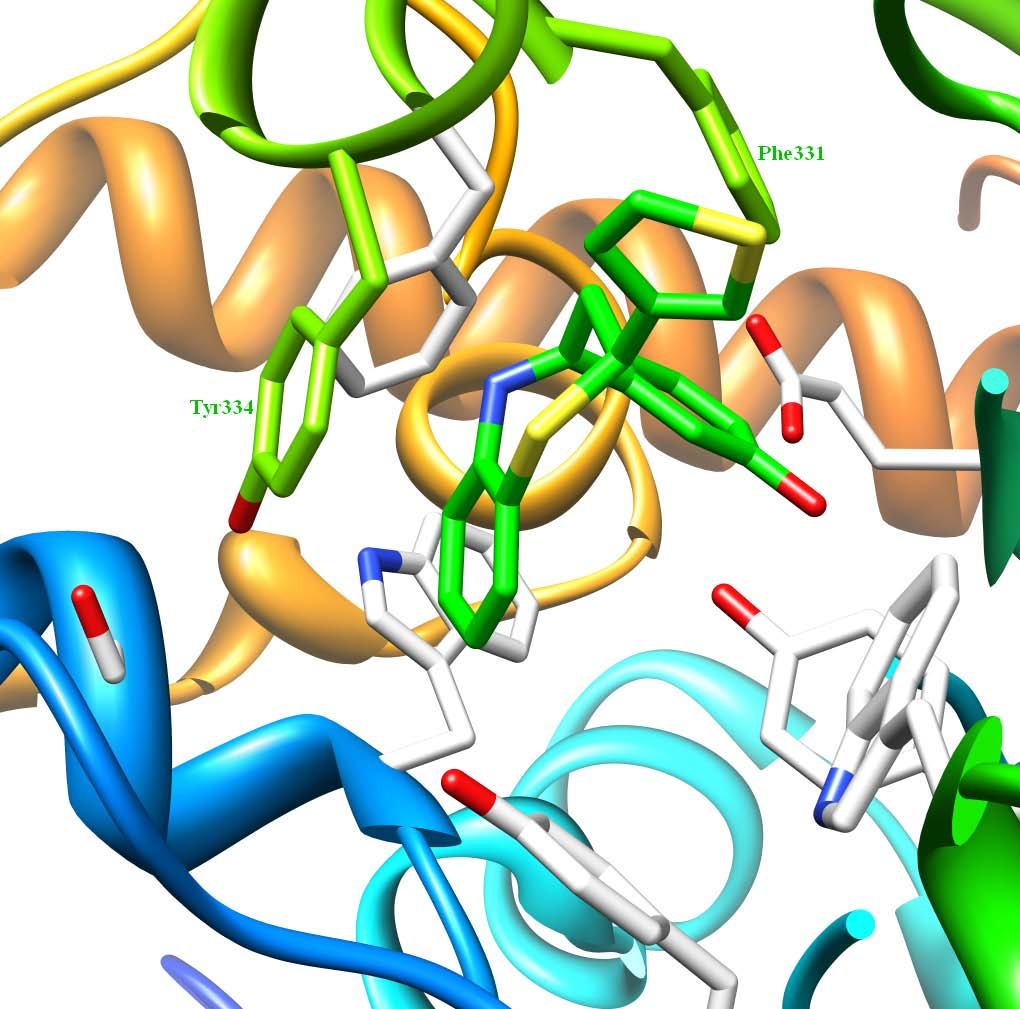
**Role of aromatic amino acid residues**. π-π interactions between the ring A of Compound A and aromatic side chains of Tyr334 and Phe331 (chartreuse). Residues are shown within 5.0 Å in the active site of AChE.

The geometrical features of the ligand-enzyme complexes were correlated on the basis of their morphology and amino acid environment in the macromolecular cavity. It was observed that π-π interactions played an important role in stabilizing the complexes. Nine amino acids - Tyr70, Ser81, Trp84, Tyr121, Glu199, Phe330, Phe331 Tyr334 and His440 - were found in the active site of AChE. Compound A was found to penetrate the aromatic cleft through the flexible six-member ring A and its entrance into the aromatic cleft was supported by π-π interaction with Phe331. The ligand-enzyme interaction is further strengthened by Tyr334, which forms a π-π interaction with the six-membered core benzothiazepine moiety (Figure 8) (see also Additional file 1, Figure S1). Further, the orientation of ring A at the bottom of the cleft was supported by two hydrogen bonds, as mentioned, with Gly119 and Tyr121.

### Compound A-BChE complex

**Figure 9:**
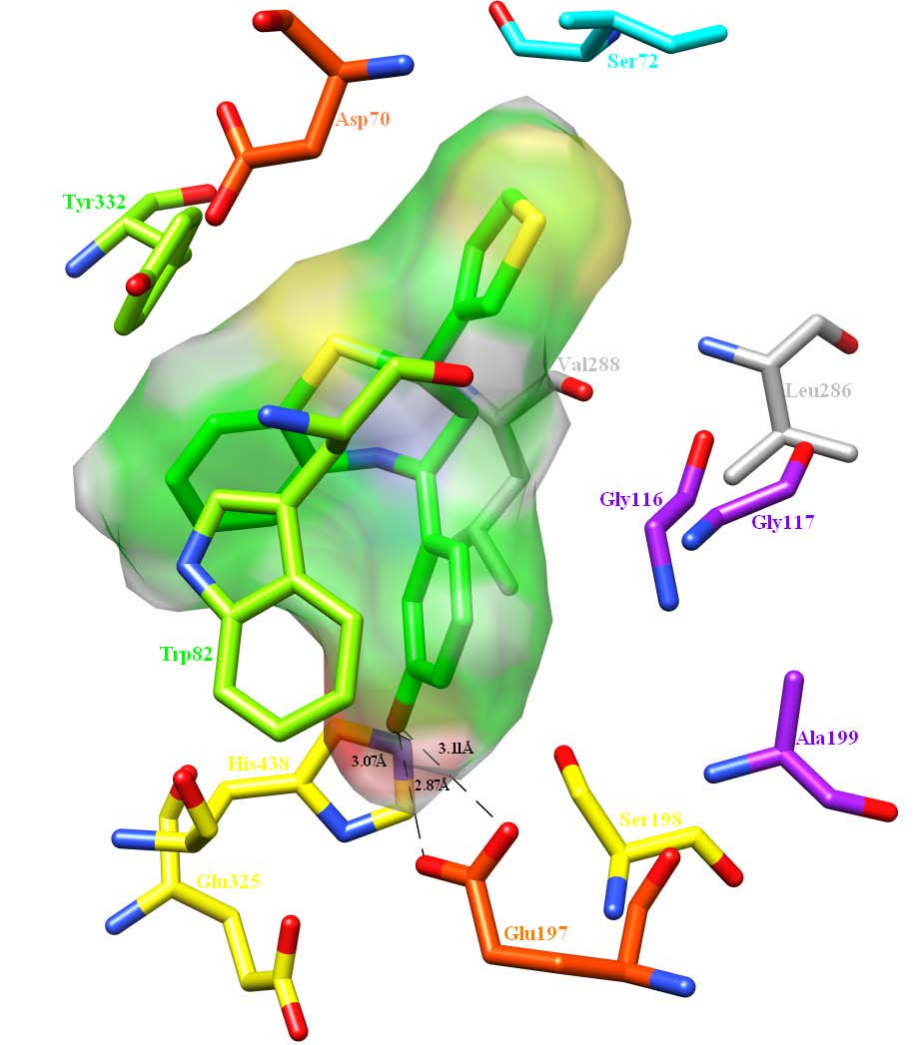
**Docked pose of Compound A in BChE**. CT (Ser198, His438, Glu325; yellow), OH (Gly116, Gly17, Ala199; purple), π-π interacting residues (Trp82, Tyr332; chartreuse) and charged residues (Glu197, Asp70; orange red) are shown here around the molecular surface representation of Compound A. The amino acid residues in grey are substituted in BChE.

Major differences between BChE and AChE are restricted to those residues that line the cleft. In the former enzyme, several of the aromatic groups of the latter are substituted by hydrophobic ones. Phe288 and Phe290 in the acyl-binding pocket (ABP) of AChE are replaced by Leu286 and Val288, respectively. These substitutions make it possible for the binding of a bulkier and more hydrophobic substrate moiety in the active site of BChE. In some cases, the conformation of the acyl binding loop (286-288) of BChE is also changed as compared to the acyl binding loop (288-290) of AChE. The catalytic triad of BChE, consisting of Ser198, His438 and Glu325, was located within the 10 Å of the docked ligand. One of the two catalytic triad residues, O γ of Ser198, is located 4.7 Å away and O ε 1 of Glu325 is located 6.9 Å away from the 3'-hydroxyl group on ring A. On comparing these distances with the distances in AChE, it is clear that Compound A is closer to the catalytic triad in BChE. However, greater potency is observed when the 3'-hydroxyl substituent on ring A makes a hydrogen bond with N ε 2 of His438, the third catalytic triad residue. The orientation of N ε 2 of His438 at an angle of 108.859º makes it suitable as a hydrogen bond acceptor at bond length of 3.07 Å. Owing to the robust bond angle of C γ itself at 119.972º, N ε 2 of His438 forms an energetically favorable environment for the 3'-hydroxyl substituent on ring A to form a hydrogen bond. The adjacent amino acid residue, Glu197 with both its carboxylates O ε 1 and O ε 2, is also involved in hydrogen bonding interactions with the same hydroxy group on ring A (Figure 9).

The catalytic triad residues of human butyrylcholinesterase (hBChE) are interconnected by hydrogen bonds. This situation is found in one of the alternate conformations of the catalytic serine in the choline-hBChE complex (PDB ID: 1P0M). The interaction with Ser198 maintains His438 in the same position as observed in all hBChE crystal structures to date. In contrast to the trivial hydrogen bonding interactions between catalytic triad, His438 is no more available to Ser198 when Compound A is present. The disruption of the hydrogen bond between O γ of Ser198 and N ε 2 of His438 does not allow efficient proton transfer between Ser198, the leaving alcohol product, and the water molecule that hydrolyzes the acyl-enzyme.

**Figure 10:**
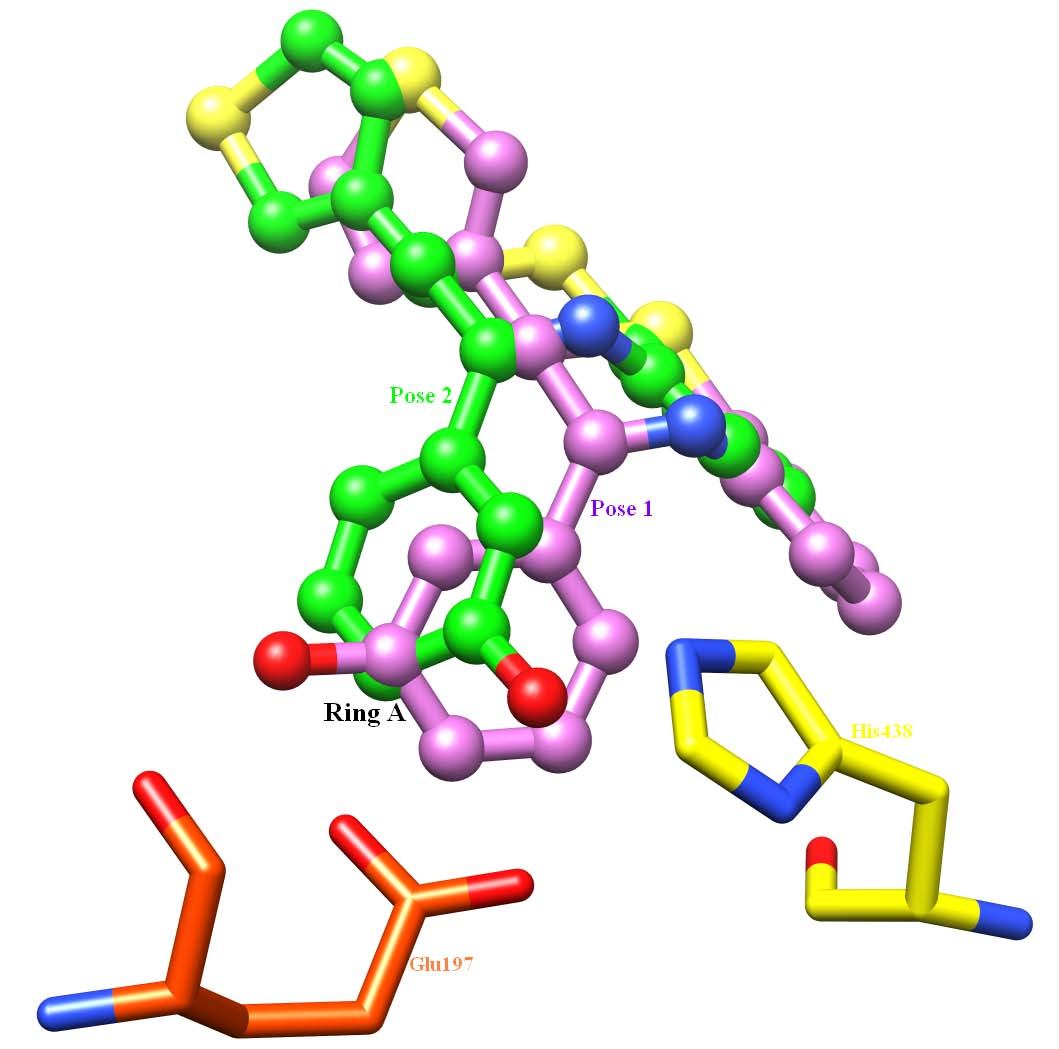
**Selection of best pose**. Two top poses of Compound A are superimposed to justify the selection of correct posture that makes appropriate interactions with BChE. The rotation around C-C bond makes ring A more prone towards hydrogen bonding (see Results and Discussion).

Another important feature of the benzothiazipine-hBChE complex is the participation of the carboxyl group of Glu197. To catalyze the mechanism of aging process, a water molecule serves as a relay for Glu197 to stabilize the positive charge on the protonated His438. Because of its interaction with Glu197, the water molecule is activated. This water molecule is ideally positioned to catalyze the mechanism of aging [51]. However, in this study, it was observed that the 3'-hydroxy substituent on ring A engaged both carboxylic oxygen atoms of the side chain of Glu197, making it impossible for BChE to catalyze the aging mechanism. The two hydrogen bonded oxygen atoms of Glu197, which serve as acceptors, differ markedly in their bond lengths and bond energies. The O ε 1 forms a hydrogen bond at bond length of 3.11 Å with energy of -0.02 kcal/mol while O ε 2 makes stronger hydrogen bond of length 2.87 Å at a lower energy value of -2.5 kcal/mol. Besides this, the flexible ring A of Compound A accounts for its potency against BChE since the position of ring A in pose 1 is flipped as compared to pose 2 owing to C-C rotation, making the pose more effective for inhibition (Figure 10). This rotation around the C-C bond brought the 3'-hydroxy group on ring A close to Glu197 and His438, thereby reducing the energy difference between the two poses (see Table 2). Furthermore, Trp82 forms a π-π interaction with ring A and the benzothiazipine moiety of Compound A while Tyr332 forms a π-π interaction only with the benzothiazepine moiety, which helps to stabilize the overall posture of the docked ligand (see also Additional file 1, Figure S1).

The additional stabilizing interactions between BChE and Compound A that make the BChE more prone to inactivity are hydrophobic interactions. The five-membered 2-thiophene moiety plays a deterministic role in the inhibition of BChE by making hydrophobic interactions with Ile69 in the peripheral anionic site.

### Molecular dynamics simulation of docked complexes

**Figure 11:**
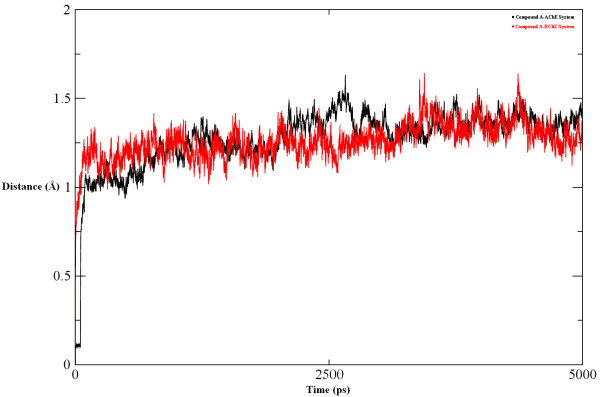
**RMSD plot**. RMSD of the backbone atoms (Cα, N, C) of 1ACL complexed with Compound A with respect to the first snapshot as a function of time.

During MD simulations, total energy, potential energy, kinetic energy, density and temperature were monitored to ensure the stability of the complex. The root mean square deviation (RMSD) values of the backbone atoms of both proteins also give information about the structural equilibration of the system (Figure 11). In the Compound A-AChE system, the RMSD increases until the 200^th ^ps and then remains unchanged up to the 2500^th ^ps. Between the 2500^th ^and 4300^th ^ps, the RMSD slightly increases once again but then decreases immediately to its initial value up to the whole simulation run time. In the Compound A-BChE system, the RMSD increases until between the 250^th^-300^th ^ps. Up to the 2800^th ^ps, the RMSD attains its maximum value but then remains stable until the end of the simulation. Overall, the Compound A-BChE system shows higher RMSD values than the Compound A-AChE system owing to the structural changes caused by Compound A. However, both systems converge towards stability and acquire equilibration.

**Figure 12:**
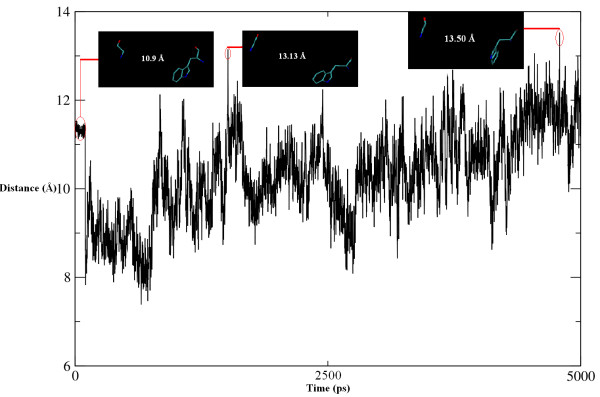
**Movement of aromatic gorge**. Width of the aromatic gorge as taken between the center of mass of Trp279 and Gly335. The increase and decrease in the width at different time intervals describes the behavior of aromatic gorge as distorted by Compound A.

Although the structure of the protein is not distinctively affected by complexation, the dynamics of the cleft are changed significantly. These dynamics have already been investigated extensively by MD simulations, suggesting that the high flexibility of the cleft is necessary for enzyme activity. A comparison of the simulation results of ligand binding with the two ChEs shows clear differences, which demonstrate that the inhibitory potential of Compound A differs among ChEs. In the Compound A-AChE complex, the extension of the cleft is vital for the entrance of Compound A. The distance between the center of mass of Trp279 and Gly335 was used to define the cleft width, which varied between 7.350 Å and 13.501 Å, with a mean of about 10.425 **± **0.1 Å. At about 225-250 ps, the increase and then decease in the width of the cleft allows Compound A to be accommodated in the active site. During the course of the simulation, the width once again increases and the cleft remains extended till the end of the simulation time (Figure 12). This may indicate disorientation in the aromatic cleft of AChE due to hydrogen bonding with the peptidic NH of Gly119 formed by Compound A.

**Figure 13:**
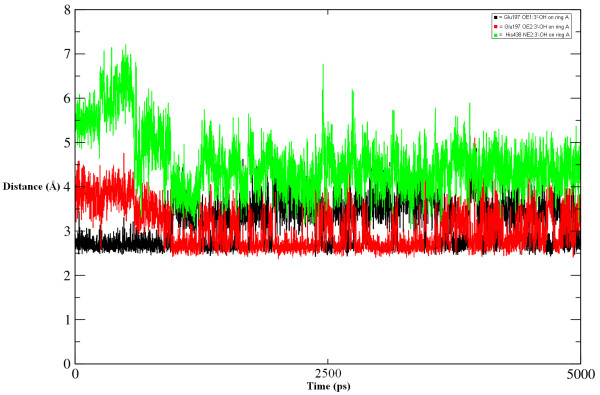
**Disturbance of catalytic triad**. Involvement of hydrogen bonding interactions during the simulation time.

The hydrogen bonds seemed to be stronger in the Compound A-BChE complex than in the Compound A-AChE complex. Figure 13 shows the change in hydrogen bonding distance between the hydrogen bonded atoms. It is noticeable that for approximately half of the simulation study, O ε 1 atom of Glu197 holds the substituent on ring A very strongly. As Compound A fits much better in the active site, it causes a constraint on the 3'-hydroxy group of ring A, decreasing the strength of the hydrogen bond for a very short time (500-625 ps) and allowing it to hydrogen bond with another atom (Glu197 O ε 2). Both these Glu197 carboxylate oxygens assist in the correct positioning of Compound A.

**Figure 14:**
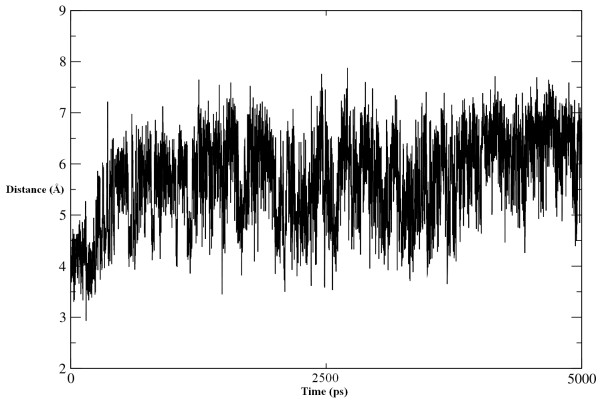
**Breakage of hydrogen bonding interactions between Ser198 and His438**.

The hydrogen bond network interconnecting the residues important for substrate binding and catalysis is highly persistent. The stabilization of this network results from a mixture of electrostatic and solvent ordering effects. Another important aspect of the inhibitory potential of Compound A for BChE shown in this study is the breakage of the hydrogen bonding pattern in the catalytic triad normally observed in BChE. This molecular dynamics simulation study illustrates that the O ε 2 atom of Glu197 makes a hydrogen bond with the ligand; the N ε 2 atom of His438 is also caught up in hydrogen bonding to the same atom of the ligand, resulting in the cleavage of the hydrogen bonding interaction between His438 and Ser198 (Figure 14). This situation makes complex of the catalytic triad which is susceptible for the natural substrate. In other words, the enzyme is unable to catalyze its natural substrate to product.

The greater flexibility of the outer residues is well illustrated by that of the conserved Asp70 residue [51]. The Asp70 carboxylate oxygens constantly break and form hydrogen bond contacts with the nearby hydroxy groups of Ser72 and Tyr332, and meanwhile, Tyr332 engages itself in a cation-π interaction with the 2-thiophene ring of Compound A. A cation-π interaction is also formed by the ligand and Phe288 of AChE, but the distance between these two rings is so large that this interaction is negligible.

## Conclusion

Current knowledge of the binding modes and the molecular interactions of the two neuronal Compound A-ChE complexes allow us to design and synthesize compounds possessing either AChE-BChE selectivity, well balanced AChE-BChE specificity or high selectivity for BChE only. The last option is more important since the content of BChE in the brain increases with age, whereas the activity of AChE declines. BChE may, therefore, play a more prominent role in ACh hydrolysis in the aging brain. The presence of BChE in the amyloid plaques and neurofibrillary tangles of AD remains an intriguing observation. It seems reasonable to assume that this enzyme is a glial product and that its location in the plaques and tangles may result from the overall inflammatory processes relating to AD.

Herein, we have reported a comparative study of the inhibition of ChEs by Compound A using molecular docking and molecular dynamics simulation techniques. The objective of this study was to provide a possible relationship between the experimentally determined IC_50 _values and the docking interactions of Compound A in the active sites of ChEs. The docking of Compound A with both ChEs has provided valuable information about the nature of the binding interactions of benzothiazepine with these enzymes. While docking algorithms often successfully predict the bound conformations of a ligand (often down to ≤ 1 Å RMSD of the experimentally determined bound conformation), most algorithms do not allow for side chain or backbone movement in the binding pocket, ignoring conformational changes that occur upon ligand binding. It was the goal of this docking and scoring evaluation to investigate as systematically and exhaustively as possible the current state of the art in docking and scoring to determine the best possible binding mode. Our suggested molecular docking protocol accounts for both situations; that is, it can reproduce a bound complex from the crystal structure considering the side chain movement. Molecular dynamics simulations illustrate the dynamic behavior of the cleft and the interactions of Compound A with both ChEs. These results provide a rationale for the greater inhibitory potential of Compound A, which was found to be primarily based on Gly119, and Glu197 and His438 in the active sites of AChE and BChE, respectively. In the case of BChE, the hydrogen bonding network interconnecting the catalytic triad residues (O ε 1 of Glu325, N ε 2 of His438 and O γ of Ser198) is highly important for substrate binding and catalysis. Our molecular dynamics simulation revealed a disturbance of this network due to the presence of Compound A. No such interactions of catalytic triad residues of AChE were observed, consistent with the low IC_50 _value of Compound A for this enzyme. On the basis of these *in silico *studies, it is possible to predict that benzothiazepine with a 3'-hydroxy group on ring A and a 2-thiophene moiety as ring B may be a strong candidate as BChE inhibitor. The fact that benzothiazepine was found to inhibit BChE more strongly than AChE may be rationalized on the basis of the observation that numerous docked poses of Compound A scored very highly in terms of hydrogen bonding. The molecular features of Compound A that are responsible for its specific inhibitory potential against BChE are the 2-thiophene moiety with an E-total of -40.815 kcal/mol, compared to the same feature in AChE (-38.412 kcal/mol). The 3'-hydroxy substitution on ring A further provides an E-total of -10.028 kcal/mol to the docked conformation in BChE, while the same feature in AChE gives -7.098 kcal/mol. Additionally, hydrophobic interactions seemed to play a pivotal role in the more specific binding of Compound A to BChE. This study may provide a rational basis for the structure-based design of benzothiazepine drugs, in combination with a selected docking protocol, with improved pharmacological properties.

## Competing interests

The authors report no conflicts of interest and they are responsible for the content and writing of the paper.

## Authors' contributions

FLA conceived and supervised the idea, WK and SK did the computational work. WK was involved in manuscript writing. ZU supervised the computational work and the manuscript editing. All authors read and approved the final manuscript.

## Supplementary Material

Additional file 1 **Figure S1 2 D depiction of key protein-ligand interactions**. A) Compound A-AChE and B) Compound A-BChE Click here for file
