# Supplementary figures and images for: In silico modeling of the specific inhibitory potential of thiophene-2,3-dihydro-1,5-benzothiazepine against BChE in the formation of β-amyloid plaques associated with Alzheimer's disease
Source: Theor Biol Med Model. 2010 Jun 16;7:22. doi: 10.1186/1742-4682-7-22 (PMC2905356; doi:10.1186/1742-4682-7-22)

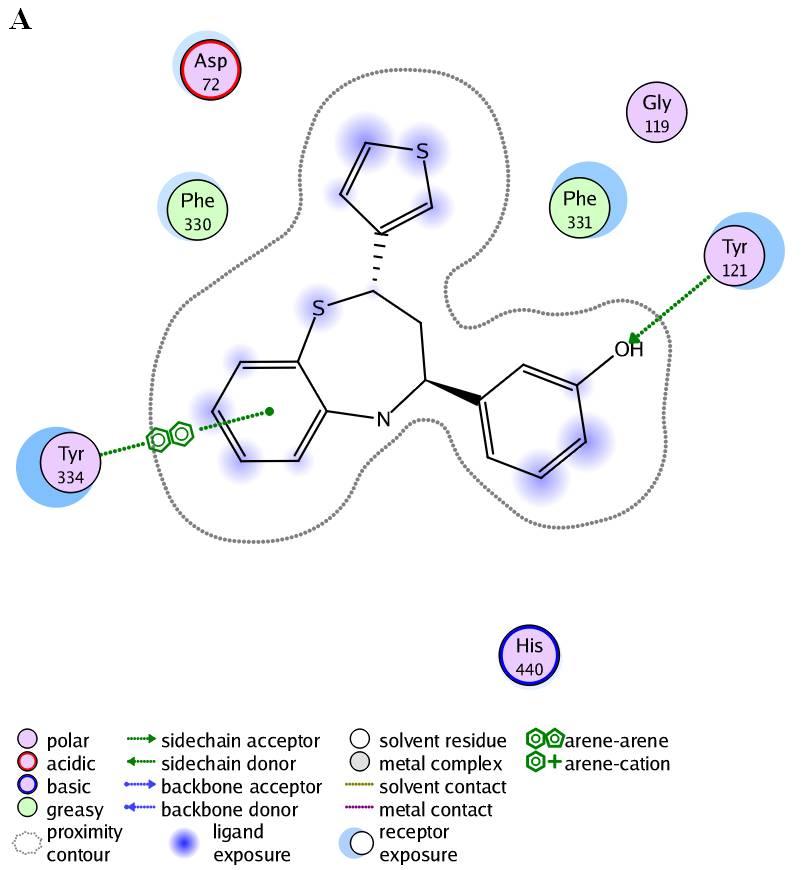


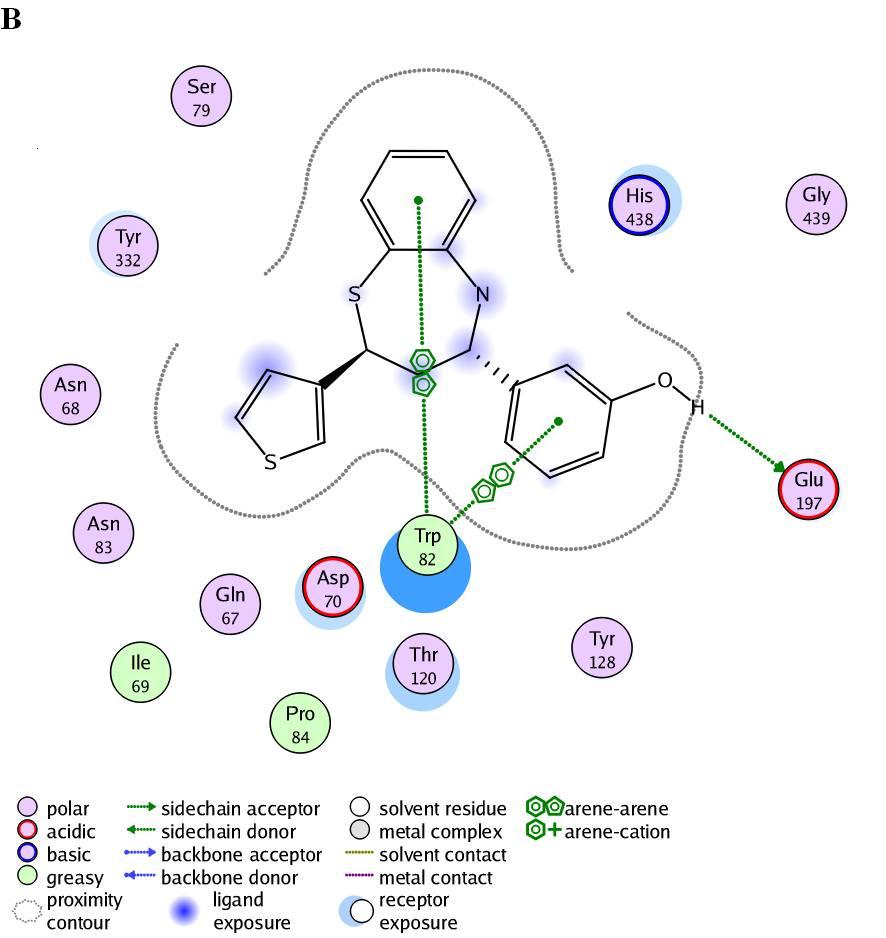

Supplement: Additional file 1 — Figure S1 2 D depiction of key protein-ligand interactions. A) Compound A-AChE and B) Compound A-BChE [file 1742-4682-7-22-S1.DOC]
